# Evaluation and learning in complex, rapidly changing health systems: China’s management of health sector reform

**DOI:** 10.1186/s12992-018-0429-7

**Published:** 2018-11-20

**Authors:** Yue Xiao, Lewis Husain, Gerald Bloom

**Affiliations:** 1 China National Health Development Research Institute, No. 38, Xueyuan Rd., Haidian District, Beijing, 100089 China; 2 Institute of Development Studies, Library Road, Brighton, BN1 9RE UK

**Keywords:** Healthcare system, Change management, Innovation, Learning, Policy evaluation, China

## Abstract

Healthcare systems are increasingly recognised as complex, in which a range of non-linear and emergent behaviours occur. China’s healthcare system is no exception. The hugeness of China, and the variation in conditions in different jurisdictions present very substantial challenges to reformers, and militate against adopting one-size-fits-all policy solutions. As a consequence, approaches to change management in China have frequently emphasised the importance of sub-national experimentation, innovation, and learning. Multiple mechanisms exist within the government structure to allow and encourage flexible implementation of policies, and tailoring of reforms to context. These limit the risk of large-scale policy failures and play a role in exploring new reform directions and potentially systemically-useful practices. They have helped in managing the huge transition that China has undergone from the 1970s onwards. China has historically made use of a number of mechanisms to encourage learning from innovative and emergent policy practices. Policy evaluation is increasingly becoming a tool used to probe emergent practices and inform iterative policy making/refining. This paper examines the case of a central policy research institute whose mandate includes evaluating reforms and providing feedback to the health ministry. Evaluation approaches being used are evolving as Chinese research agencies become increasingly professionalised, and in response to the increasing complexity of reforms. The paper argues that learning from widespread innovation and experimentation is challenging, but necessary for stewardship of large, and rapidly-changing systems.

## Introduction

An increasing body of research attests to the complexity of health systems, and argues that managing change requires approaches that take into account system complexity, context and implementation processes [1, 2]. Over the reform period, from the 1970s onwards, Chinese policymakers have encouraged sub-national innovation in many policy areas as a means of exploring practical and innovative approaches, with the hope that learning from widespread and often relatively unscripted experimentation will help guide the reform process. As a corollary to this process of eliciting innovative practices, mechanisms are needed that can help central policy makers identify and understand emerging practices, their potential usefulness, and the extent to which they can or should be propagated. This kind of process confronts the question, posed by Dani Rodrik, “We shall experiment, but how shall we learn?” [3]. This is a particular challenge for China, given its size, the multiple ways that implementing jurisdictions may vary, and the limits to knowability of what may work, how, and why, in advance of actual implementation.

In this article we use case examples to show the role of a government think tank, the China National Health Development Research Centre (CNHDRC), in supporting system-level learning from dispersed policy practice in the context of rapid change. It explores how interactions between researchers/evaluators and decision-makers at various levels can ensure that studies contribute to effective policy steering.

## Evaluating health policy implementation and innovation in China

### Health policy implementation and innovation

Sustained adaptation since the beginning of market-oriented reforms in the late 1970s has transformed China from a poor, predominantly rural, country into an increasingly urban society, with a larger and more diverse economy, higher incomes and improved nutrition, health and welfare indicators [4]. China’s government system combines central government leadership with highly localised policy implementation and management of reforms [5]. While policy implementation in China has been studied for many years, the study of policy experimentation and innovation has recently received increasing prominence as a possible explanation of China’s ability to manage large-scale system adaptation over time and improve population welfare [6, 7].

China’s government system spans many levels, from central government and line ministries (including agencies responsible for health) through provinces, cities, counties, towns/townships and villages. Cities and counties have a very important role, despite their relatively low position within the government system, and many decisions are decentralised to these levels, including much day-to-day planning and management of health and welfare systems [8]. In many respects, this is simply a reflection of the challenges of managing a vast system: China has almost 3000 counties, and a large number of cities, which vary a great deal.

Decision makers have made a lot of use of experimentation and innovation in the management of reforms, including in the health system. This article uses a vocabulary of ‘reform’ to underline the institutional nature of processes underway – reforms to the health system require adjusting the roles and behaviours of a wide range of institutional and individual actors to advance towards hoped-for outcomes, such as a better functioning health or welfare system in a context of a rapidly changing context [9, 10]. Relatively controlled experiments may be combined with use of ‘open’ policy frameworks by central/provincial government in multiple rounds of change to foster adaptation, learning by doing, and innovation [11]. Faced with the pressing need to reform and adapt, this approach has the potential to reduce the risk of large-scale policy failure.

‘Experimentalism’ has roots in China’s early twentieth century experience, but has been commonly used since the beginning of market-oriented reforms in the 1970s [12]. It is hoped that experimentation and the fostering of innovation can engage the initiative of sub-national governments and departments in widespread problem-solving, although a degree of implementation failure and deviation is an inevitable side-effect [13]. In the health sector, the Chinese government has frequently adopted an approach of “experiment, experience and expand” (3E) to allow local governments discretion in policy adaptation, reform design and innovation [14, 15] and legitimise and encourage local initiative within certain, often loosely-set, parameters [11].

The Chinese policy community employs an identifiable discourse of innovation, which characterises innovation as the use of new approaches to disrupt existing states, systems or patterns of behaviour to create new, *emergent* patterns and rules that can “sustain public welfare and properly motivate key stakeholders” [16]. This discourse also helps define the appropriate roles of central and local governments, in which central government defines the principles and foci of reforms, and local governments act as hands-on experimenters/innovators [12], and enables signalling of emergent and potentially useful practices [5].

### Evaluation as a decision-supporting tool in health system reform

Much has been achieved in reforming the Chinese health system, but much remains to be done. A recent report by the World Bank, WHO and the Government of China argues that more robust and systematic mechanisms are required for gathering information and learning to inform ongoing reforms [17]. The ‘open’ nature of many reform processes, the high degree of discretion given to local actors and the great range of starting points and possible dynamics in implementing jurisdictions, create challenges for the central government not just in understanding implementation successes/failures but, more profoundly, in understanding and learning from new/emergent institutions and practices that arise through this kind of semi-structured reform process. Learning from emergent practices becomes an important probe of how reforms are progressing. Use of a label, such as ‘innovation’, is part of a signalling process of identifying emergent practices that may have value at a system level.

This great variation in emergent practices creates a need for mechanisms that can help policy makers identify and understand them and assess whether they can or should be promoted or propagated. There is a need to screen good practices from a range of emergent policy practices [18], uncovering ‘positive deviants’ (better than average practices in any given cohort) [5], and assess the extent to which specific practices or innovations are likely to be of relevance, or reproducible, in other jurisdictions and therefore worth propagating – a question of external validity [3, 19]. As Wagstaff et al. observed in the context of China’s rural health insurance programme, “the policy of ‘letting a thousand flowers bloom’ … has much to commend it in terms of encouraging innovation, but it makes pinpointing the secrets of success very hard” [20].

The Chinese government has historically employed a number of practices and ‘informational infrastructures’ [21] to promote intra-systemic learning, including research institutes with a mandate to provide policy-relevant research.^1^ The CNHDRC, formerly the National Health Economics Institute, is an example. It was established by the Ministry of Health in 1991 as a government think-tank. Its functions have evolved over time and in 2007, it set up a dedicated health policy evaluation and technology assessment unit. This has conducted a wide range of evaluations of pilot programs and policies, such as implementation of clinical pathways and payment reforms, medical pricing reforms, and the 12th Five Year Plan for Health. The CNHDRC has a mandate to inform national decision-making. Through ten years’ experience, the CNHDRC has developed approaches to evaluation closely linked to the Chinese institutional context. The proximity of the CNHDRC to stakeholders at both central and local levels has led to an understanding of the need to build relationships with key stakeholders and to a focus on utilisation, and end users, in its research and evaluation work.

The following cases demonstrate how evaluation of new policies/reform initiatives has supported communication between multiple stakeholders, and facilitated system learning through intensified evaluator and end-user interactions. Looking retrospectively, approaches taken by the CNHDRC evaluators have resembled ‘utilization-focused evaluation’ [22]. Besides supplying hard evidence on implementation effectiveness, the CNHDRC evaluators’ role has included that of facilitating learning and adaptive management approaches.

## Case studies

This section describes two policy implementation studies carried out by the CNHDRC in which local governments had a lot of discretion and space for experimentation, to show how utilization-driven evaluative thinking was used as a change management tool. Both studies were commissioned by the central health authority with the aim of understanding progress in implementation (successes and failures) and to learn from new/emergent institutions and practices arising in jurisdictions carrying out the reforms as a guide for national policy making. The investigators framed research questions to meet decision-making needs and also worked with the intended users to visit the pilot sites, allowing them to directly observe practices, and creating a platform for ongoing interaction between the evaluators and decision makers. This approach helped to uncover problems of implementation, and foster learning and information-sharing among key stakeholders, especially between local implementers and central policy-makers.

In both cases, the evaluators used multiple methods to probe new and emerging practices and uncover the factors influencing their direction of development. This was combined with attempts to assess the effectiveness of these practices by employing quantitative methods such as cost-effectiveness analysis. Information channels and feedback loops were formed, which helped inform subsequent stages of policy-making. The following sections profile CNHDRC’s evaluations of the reform of China’s rehabilitative care delivery system.

### Study of pilot work on integrated rehabilitative care delivery in 7 pilot cities

#### Background

In 2009, the Chinese government launched radical national health system reforms aimed at establishing an integrated healthcare system providing preventive, curative and rehabilitative care. Rehabilitative care has been underdeveloped in the Chinese healthcare system for decades, with shortages of human resources and fragmented care delivery. During 2011–2013, the Ministry of Health launched experimental pilots in 46 cities aimed at improving the delivery of medical rehabilitation. It issued a guiding plan, delineating the basic principles and identifying the main elements of the reform, but it left detailed design to local governments [23]. The MoH commissioned the CNHDRC to conduct an evaluation to inform policy formulation and it sent a supplementary policy document to all pilot sites on evaluation requirements, with a detailed plan for monitoring and evaluation.

In consultation with decision-makers, the CNHDRC team produced guidelines for monitoring and evaluation (M&E) before the launch of the pilots. The M&E aimed to measure progress, identify problems, facilitate adjustment of the rehabilitative care system, and extract workable models, approaches and mechanisms to inform subsequent policy development and scale-up. The M&E was conducted in 2 stages. During the first stage (2011–2012), the evaluators collected quantitative and qualitative data to review progress in each locality and visited key sites to identify issues and summarize lessons and experiences. During the second stage (2013), seven pilot cities were selected for more detailed study, including a cost-effectiveness analysis that compared pilot and control hospitals.

Only two of the seven pilots showed the anticipated impact. Since the pilot program represented 3 years’ of effort by local governments and pilot hospitals in 46 cities, the central decision-makers did not want to draw hasty conclusions based on these results. They wanted to know why some pilots could demonstrate impact while most others could not. Following discussion with the decision-makers, the CNHDRC researchers shifted their focus to studying the mechanisms leading to certain outcomes in each locality, in an attempt to explain the cause of failures or successes.

**Fig. 1:**
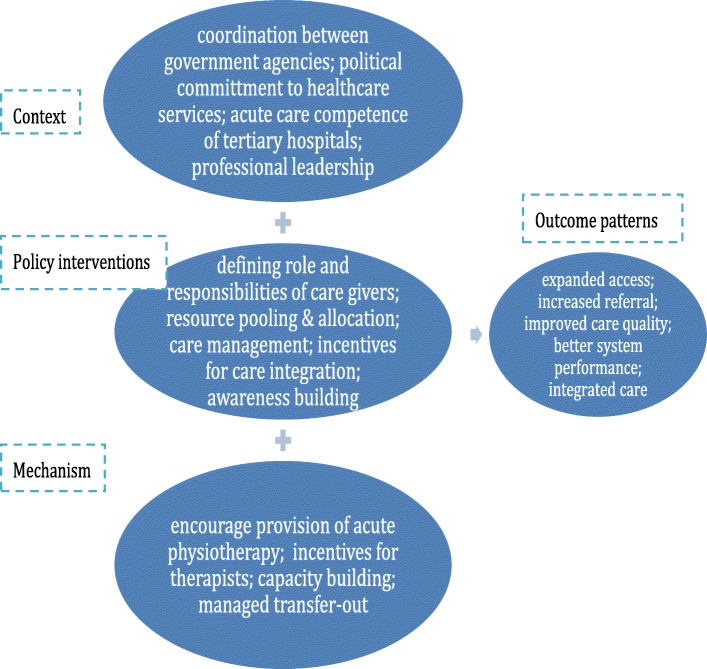
The middle-range theory of the national pilot program

The researchers drew on the approach of realist evaluation to design this study. They made use of a ‘context-mechanism-outcome’ (CMO) framework to develop case studies showing what works for whom in what context [24]. By reinterpreting the data and findings, they uncovered interesting stories about how some localities achieved the goal of the reform. Mechanisms chosen by different local reformers in specific contexts were studied and common patterns of local choices were identified to form a middle-range theory (Figure 1) for understanding the mixed results of the impact analysis.

#### Different approaches to local reforms

The approach to piloting was experimental to allow local authorities space to develop innovative practices that could inform national policy. City governments showed three types of behaviour: passive incorporation of national policy requirements into local implementation plans; reflection of national policy in local implementation plans, but with tweaks to reflect the local context; and development of innovative local reforms to re-interpret or expand national policy in an attempt to meet local needs in a contextually-specific way. The evaluators were most interested in cities that took the third approach, since they were most likely to generate innovations or breakthroughs that could advance national policy making.

The approaches taken varied, due to the choices and strategies of local stakeholders (including policy-makers, program managers, and service providers) and the reactive behaviours of key stakeholders in the pilot cities (including health managers and medical staff in local health facilities). The reforms stimulated a range of reactions by key stakeholders, most notably the health care institutions. Some were passive, while others viewed the reform as a chance to secure more resources and policy support. Hospital presidents and department heads were key decision-makers, and put in place many measures to promote care synergy, expand access, and encourage utilization of care. Hospital managers commonly used economic incentives to encourage provider behaviour change. Some active reformers stood out from the rest, and contributed to further development of the reform.

Politicians and health policy-makers in localities with successful pilots tended to play a critical role by coordinating and streamlining policies concerning care planning, service delivery, pricing and payment of rehabilitative services. Without political and institutional backup, facility-level success could hardly have been replicated. Pioneering policy-makers expanded the reach of the policy by exploring synergy between different policies concerning rehabilitative care. This provided a favourable context for nurturing reforms. For example, Shanghai Municipality coordinated relevant agencies, such as the Bureau of Human Resources and Social Security, and the Municipal Disabled People’s Federation. A joint policy on transforming some secondary public hospitals into rehabilitative care facilities was issued, and three hospitals were transformed into specialty care facilities by the time of the CNHDRC investigation, greatly increasing overall rehabilitation capacity.

Resource pooling was also a common policy measure. Kunming, Shandong and Beijing pooled resources from the Disabled People’s Federation to support delivery of medical rehabilitation. Kunming Municipality encouraged pilot hospitals and rehabilitative practices owned by the Disabled People’s Federation to share practices and funding and to deliver care for both acute and long-term patients. The Bureau of Human Resources and Social Security manages funds for occupational rehabilitation care for enrolees of occupational injury insurance schemes, and usually designates hospitals or wards for its patients. However, no particular mechanism was introduced to change the benefit package of the publicly funded health insurance schemes, and huge disparities of coverage and benefits for the patients covered by occupational insurance and others remained in all pilot sites.

Against the background of ongoing public hospital reform, the pilot hospitals mostly adopted clinical pathway management, and tried to adjust care pathways by including rehabilitative care. Common institutional approaches to care integration included incentives for timely and active acute rehabilitative care, and managed discharge of patients. Huashan Hospital in Shanghai sent therapists to work in neurology and orthopaedics wards to promote bedside physical therapy, which shortened the average length of stay (ALOS) in these wards. Hospitals in Kunming, Harbin and Zibo closely managed discharge of patients, to ensure continuous care.

Some innovations, aimed at addressing particular challenges, stood out. In order to improve utilization of rehabilitative care, township health centres in Zibo city used acupuncture and TCM medicines as part of rehabilitative care pathway for stroke patients. In Liushui Township Health Centre of Zibo, informed consent was obtained from patients who refused to undertake rehabilitative care to ensure they were aware of the risks of giving up important rehabilitative care. Such innovative measures were usually taken under the leadership of strong policy-makers or care managers.

#### Factors contributing to different outcomes

The researchers undertook a preliminary mapping of contexts, mechanisms and outcomes [17] to describe reforms underway in each pilot city. Table 1 summarises the findings on several successful and unsuccessful pilots. This allowed the evaluators to summarise common mechanisms and emergent practices, to *probe* [25] the operation of national policy in the actual institutional contexts encountered in the pilot cities, and then provide feedback on the operation of policy in the field to national policy makers.

**Table 1:** Outstanding CMOs in the 7 pilot cities

Pilot Cities	Main policy interventions	Contexts + Mechanisms + Outcome patterns
Beijing	Pilots covering all city districts; defining role and responsibilities of care givers; resources pooling; encouraging public-private partnership (PPP); incentivizing timely and continuous care.	- **C:** previous trials on rehabilitation care delivery; strong political and professional commitment; strong financing capacity; public tertiary hospitals packed with patients; limited acute rehabilitative competence in tertiary public hospitals. - **M:** promoting bedside acute rehabilitation; commissioning care from competent private rehabilitative hospitals; capacity-building on long-term rehabilitation; managed transfer-out. -**O:** improved accessibility and effectiveness, proved cost-effectiveness;
Shanghai	Pilots in 2 city districts; defining role and responsibilities of care givers; increasing rehabilitation resources and input; care coordination.	-**C:** political commitment; good cooperation between government agencies; public tertiary hospitals packed with patients; strong acute rehabilitative competence in tertiary hospitals; inadequate rehabilitation resources. - **M:** competent tertiary hospitals taking lead in building service network; encouraging training and staff exchanging to build up post-acute rehabilitation capacity in secondary and primary care; rehabilitative resource planning; financial incentives for therapists; managed transfer-out. - **O:** improved accessibility, affordability and effectiveness, no evidence for cost-effectiveness.
Harbin	Initial pilots in the major teaching tertiary hospital and its hospital alliances; defining role and responsibilities of care givers; optimizing resources allocation; incentivizing care integration and referral.	- **C:** good cooperation between the municipal health authority and disabled people’s federation; attention to regional rehabilitative capacity planning; strong acute rehabilitative competence in tertiary hospitals. - **M:** contract-based cooperation of rehabilitation care providers; encouraging training and staff exchanging to build up post-acute rehabilitation capacity in secondary and primary care; incentivizing therapists in the tertiary hospital for providing care in secondary and primary care facilities; managed transfer-out. -**O:** improved accessibility and effectiveness; proved cost-effectiveness.
Zibo	Pilots in all health facilities; defining role and responsibilities of care givers; leadership development; resource pooling; stress on use of TCM; public awareness building;	-**C:** strong political commitment; multi-agency cooperation; development of traditional Chinese medicine; payment-based referral incentives. -**M:** clinical protocol and guidance development; intensifying capacity building on post-acute rehabilitation care; financial incentives for therapists; managed transfer-out. -**O:** improved accessibility and effectiveness, no evidence of cost-effectiveness.
Changsha	Initial pilot selected hospital; defining role and responsibilities of care givers; encouraging PPP; increasing service provision by private sector; resource pooling.	-**C:** strong medical rehabilitative capacity; political commitment in developing public-private partnership; acute rehabilitative competence in tertiary public hospitals; well-developed private sector. -**M:** pooled resources for developing a private tertiary rehabilitation center; contract-based cooperation of rehabilitation care providers; managed transfer-out. -**O:** improved accessibility of rehabilitation, but no evidence for cost-effectiveness.
Kunming	Initial pilot in a care alliance set up by the largest teaching hospital; defining role and responsibilities of care givers; resource pooling.	-**C:** Pre-existing collaboration between the Provincial Disabled People’s Federation, the Medical Rehabilitation Association and the pilot teaching hospital; strong clinical leadership; incentivizing therapists for providing timely acute care and supporting long-term care in community health centers; acute rehabilitative competence in tertiary public hospitals. -**M:** incentivizing therapists for providing timely acute care; contract-based care coordination and integration; efficient performance management; stress on pathway-based management and quality improvement; managed transfer-out. -**O:** Improved accessibility and effectiveness; proved cost-effectiveness.
Urumqi	Initial pilots in competent health facilities; defining role and responsibilities of care givers.	-**C:** Strong professional commitment; inadequate financing and payment policy support; strong care coordination capacity of the pilot hospitals; acute rehabilitative competence in tertiary public hospitals. -**M:** care alliance initiated by the pilot teaching hospital; financial incentives for bedside acute rehabilitative care; technical support for facilities providing post-acute rehabilitative care. -**O:** improved accessibility; no evidence for cost-effectiveness.

Many novel local practices were assessed during field visits by the research team, and comments and recommendations were fed back to local decision-makers. Field visits carried out by the research team usually included central health policy-makers, health policy or management experts, and clinical experts, providing a solid basis for conducting rapid assessments. Focus group discussions with local stakeholders were used as an informal platform for discussing problems and potential solutions, and opened up a space for information sharing and learning. In many cases, this helped build consensus among different actors, promoted a common assessment of new practices, and dissemination of local innovations. In some cases, new and innovative practices received positive feedback and were fed into further decision-making. For instance, after the first phase of piloting, Shanghai issued a policy to turn several secondary public hospitals into rehabilitative hospitals to strengthen rehabilitative care capacity, and this novel reform has continued to be built on in the care integration reform in Shanghai, following which some underperforming public secondary hospitals have been transformed into rehabilitative hospitals to strengthen their rehabilitative and long-term care capacity.

Based on a comparison of the strategies and measures adopted by key agents in different localities under the middle-range theory of the pilot, the researchers formed policy recommendations for further development of the national pilot program. Political commitment, cross-governmental cooperation, acute rehabilitation competency in tertiary hospitals, and professional leadership were identified as key contextual factors for successful implementation. Defining roles and responsibilities of different care givers, effective resource pooling and allocation, incentives for care coordination and integration were common effective policy interventions used by the pilots and the potential for scaling up was discussed. The report stimulated further discussions between the MoH and other ministries regarding rehabilitative care, and the end of 2016 saw issuance of a joint policy by the MoH and Ministry of Human Resource and Social Security, under which more rehabilitative care was included in the national benefit package to provide financial incentives for care coordination and integration, and some core recommendations of the report were referenced.

### Evaluation of the merger of maternal and child health and family planning services

#### Background

In early 2013 the Ministry of Health and the Population and Family Planning Commission were merged to create the National Health and Family Planning Commission (NHFPC). The policy on merging the two ministries was issued at the end of 2013 and by the end of 2014, 31 provinces/municipalities had completed this administrative reform at provincial level, and 17 provinces had initiated the merger of maternal and child health (MCH) and family planning facilities at city, county, and township levels. The CNHDRC was first commissioned by the Department of Health Planning and Information of the MoH, with support from UNICEF, to evaluate the merger of MCH and family planning facilities in three western provinces prior to the formal administrative reform in 2013. They produced a two page briefing for the MoH, which identified a number of potential problems, such as the possible mishandling of public property and the weakening of MCH or family planning service delivery capacity at county, township and village levels. Two ministers made comments on the briefing and requested that the Department of Maternal and Child Health organize follow-up studies on the reform.

The CNHDRC was asked to conduct a rapid assessment of progress in implementing the merger. Central policy-makers expected to see the emergence of a range of innovative practices. The merger policy evaluation had a strong utilization focus. The policy-makers wanted to follow the initial reaction and immediate results of local reforms through a more objective lens they called evaluation.

#### Study design

The merger was an entirely new initiative, and central policy provided only a general framework for local implementation. Local governments were expected to design operational plans incorporating implementation requirements, and most provincial governments conducted their own pilots before developing provincial implementation plans. There was a widespread need for learning, and the evaluation was designed as a channel for learning and sharing information. A case study design was chosen to generate knowledge about the local reform processes [26] and four rural counties from different regions were selected as typical cases. Administrative and institutional data were collected and focus group discussions with representatives of provincial, municipal and county health and family planning authorities were scheduled, as were semi-structured interviews with managers and staff working in county-level MCH and family planning facilities. The field visits were structured to enable the evaluators to collect information on emergent policy innovations, identify problems and deviations from central policy, and help local stakeholders reach consensus on key issues involved in the merger. Other relevant government agencies were also invited to participate in the provincial and county meetings, to collect their views of, and attitudes towards, the reform. The deputy county mayor in charge of health and family planning issues in each county was invited to preside at the county-level meeting.

The NHFPC arranged for four policy experts to participate in the field visits. These experts were local decision-makers with a lot of experience of working on health and family planning issues. One had participated in the design of the current reform. The aim of involving these policy insiders was to sensitize the evaluation to local contexts, filter information and innovations, and make findings more useful for their primary intended users —central decision-makers.

Given the early stage of the reform, many policy-makers at county, city and provincial levels hoped to learn from the rapid assessment by the independent evaluators. The team visiting the counties was usually joined by one or two provincial or municipal officials, who hoped to gain a deeper understanding of changes taking place at the local level and to get feedback on these changes from national experts. Deputy county mayors often attended focus group discussions of county policy-makers to present their views and concerns, and ask for more financial and policy support from provincial and national governments. The involvement of these secondary users of the evaluation findings pushed the field visits to become more utilization-driven, with a clear focus on learning.

#### Local adaptation and feedback to policy-making process

The analysis of routine institutional data revealed little about the initial results of implementation. However, interviews and focus group discussions were information-rich and insightful. The four counties showed varying degrees of progress with the reform. A range of non-linear, self-organizing and emergent behaviors of local agents were found.

Policy makers in the different localities carried out the reform in different ways. In places that had completed the administrative merger between the health bureau and the family planning commission, health policy makers tended to focus on the merger of MCH and family planning facilities, and were active in implementing the reform. In localities where the administrative merger had not been completed, health bureaus were mostly inactive in implementing facility-level reform. County governments and government agencies reacted differently to the reform, and some county governments twisted the policy to meet their own ends. For example, one county government used the reform as an opportunity to build a women and children’s hospital. This created resistance amongst staff of the county MCH Centre, whose benefits would be adversely affected if they were required to transfer to the new hospital. These staff, with encouragement from the county health bureau, complained openly and sent a signed petition to the CNHDRC researchers. After consultation with the policy experts and central health decision-makers, the researchers treated this case as a mishandling of institutional merger and reported the unintended consequences and flagged these illegitimate practices.

The report to the NHFPC noted that actors engaged in the reforms interpreted central policy differently and had varying responses to it. The evaluators found that the trajectories of the same reform in different localities were non-linear, and that they showed self-organizing and emergent behaviours by multiple agents as well as feedback loops. The ways that individual agents or institutions reacted to the reform could change its course in a given locality. Earlier reforms and local contexts were not uniform in the pilot counties, and had a large impact on how the reforms progressed, and the outcomes of the reform, in each locality. Through their role in assessing progress, the team helped to facilitate communication between policy-makers at various levels by explaining subtleties of policy to local officials and rapidly reporting local innovative approaches. In their field trips, the meetings with key reform stakeholders (such as decision-makers, policy implementers, service suppliers, etc.) at local and provincial levels played unique roles in facilitating this kind of communication. Novel and emerging practices were usually rapidly screened by the evaluators and reported either formally in their field trip reports or informally through meetings with central policy-makers. In the second case, the central government dispatched a number of individuals close to the policy process to join the evaluators, to help them rapidly arbitrate over illegitimate practices and legitimate policy innovations. In the first case, the evaluators were engaged in designing and delivering the policy experiment, to get a deep and accurate understanding of the trial and expected outcomes.

In some cases, the evaluation team was able to deliver key policy messages to localities on behalf of central decision-makers, and thereby encourage a number of good and innovative practices, and discourage illegitimate and negative practices. In some cases, the policy experts on the team were able to propose potential solutions to some problems encountered in the localities, while the presence of the evaluation team also allowed messages from the localities to be transmitted to provincial and central policy-makers. This two-way information flow contributed to mutual learning between policy implementers and designers, and helped health reformers in central and provincial government to navigate the complexity of this reform.

### The role of evaluation in screening for innovative practices and guiding reform

In the two cases, central government used open or semi-open policy frameworks to provide space for sub-national governments, and implementers designed and implemented local plans which both conformed to the principles for reform set by central authorities and spoke to complex local conditions. This ‘one size does not fit all’ [27] approach creates dynamic processes. Chinese reforms are frequently hurried, and pilot localities are given limited time to effect change. In such a situation, evaluation of policy implementation usually has a strong utilization focus. A research team trusted by the government, such as CNHDRC researchers, can form a working relationship with the intended users. This kind of early-stage evaluation of new reforms in a complex system can provide a ‘probe’ for local practices, increasing understanding of system dynamics as revealed by the reform process, and evaluators can screen for emergent innovations, both “good” and “bad”. It is important to pick up deleterious outcomes early to avoid big deviations from the intended direction of development. Narratives that can clearly tell local reform stories, and make links between context, emergent mechanisms, and (where possible) outcomes, are persuasive tools in guiding reforms. The final output of the second commission was a two-page report on key findings and policy recommendations.

The participation of ‘policy experts’ in the evaluation meant that the evaluation team also disseminated information on the core principles of policy and provided guidance to local level implementation. This, in turn, generated feedback from local implementers, which could be fed back to central decision-makers. In some cases, this informed subsequent rounds of policy making or led central authorities to introduce additional regulations. In this way, the evaluation process created a platform for learning and exchange of information between different agents or parts of the policy system. This approach served as a support to local and central reformers charged with managing change under conditions of great complexity, and helped reduce the risk of making major policy mistakes.

While such an approach helps support the management of change at scale in a rapidly changing environment, and is relatively quick and easy to conduct, it has several prerequisites. Firstly, the pilot program or policy is undergoing evolution and development, which means that there is no predefined model or best practice to copy, therefore, the purpose of initial evaluation is to rapidly screen for emerging successful practices or promising innovations, to support continuous learning and improvement of the local trials, and to feed back to program and policy development.

Secondly, evaluators must be trusted by policy-makers. As shown in the two cases, the presence of policy experts in the evaluation team increased the team’s credibility and helped facilitate dialogue between decision-makers at various levels. In the Chinese case, distinct and institutionally-specific cultures of credibility/legitimacy help underpin effective evaluation and learning. These are not solely related to technical competence in evaluation methods, but also to situatedness and contextual understanding, which we have argued to be of importance in understanding change in complex systems [27]. To an extent, the role of evaluators is not confined to that of impartial and disinterested scientists. Rather, they are actively involved in steering local reform efforts.

A third prerequisite is adequate representation of different stakeholders and systemic interests in the evaluation process. In the case of the reforms discussed here, it was important that focus group discussions with local policy-makers be arranged so as to include the main policy making agencies, including local government, health bureaus as and other government agencies with a stake in the reform, as well as other institutions affected by the reform, including health managers, medical staff and so on. Only in this way could the range of interests affected by, and impinging on, the reform be represented, and key responses to the policy be understood and reflected to the designers of the reforms.

## Conclusions

The paper has argued for the importance of a utilization-focused approach to evaluation in China’s management of experimental reform processes. The policy system in China is characterised by high levels of discretion on the part of implementing units (sub-national governments, local health bureaus, hospitals, and the like) which are on the front line of management of many health reforms. As demonstrated by the two cases, close evaluator-user interaction plays an important role in quickly screening effective local practices, engaging various stakeholders, and enabling iterative systemic learning. We argue that this is one part of ‘managing for emergence’ in complex systems, and has the potential for exploring ways to ensuring external validity. The case studies show changing demand from national policy makers, and how the Chinese research institutes and researchers are experimenting with new approaches in the evaluation of complex reforms, while building on indigenous assessment repertoires made use of by the Chinese state.

Many evaluation approaches focus on making claims about the internal validity of a given intervention, asking how sure we can be that a given action or input led to a given result. However, for managing rapid change in a complex context, Chinese decision-makers seem to be more interested in engaging researchers/evaluators at an early stage of implementation to feed into the policy-making cycle as demonstrated in case 2, or understand common patterns of local reforms and narratives behind success or failures as in case 1. We have argued that such a utilization-focused approach can expand the scope for learning through evaluation in complex systems, where the aim is to foster desirable emergent states. Assessing novel policies and practices and their potential systemic usefulness requires making claims for external validity – where contextual factors differ in many (and often unknowable) ways, how can we make claims regarding the replicability or systemic usefulness of novel practices we observe? How should we arbitrate over the distinction between innovations that deserve to be encouraged or promoted, and unhelpful or illegitimate practices that should be discouraged?

While by no means all reforms receive this degree of attention from policy-makers and evaluators, and while approaches discussed in this paper are a work in progress and need more careful study, this paper has shown how Chinese decision makers’ cultures of decision-making have led to the use of evaluation as a way to support the management of reforms in rapidly changing and complex contexts. The paper has shown how Chinese researchers are experimenting with new approaches to policy studies, attempting to better link contexts and outcomes, and how rich reform narratives, including assessments of innovative mechanisms and explanations of failures, are becoming important parts of the Chinese repertoire in attempting to systematise learning from pilot reforms. Such approaches are gaining ground in health policy research and evaluation, though more careful studies are needed to examine their linkages with existing evaluation approaches such as realist evaluation or utilization-focused evaluation.

The cases discussed here show evaluations that were designed at the outset of national pilot programmes and indicate a degree of institutionalisation of evaluation and learning in the reform/piloting process. Strong government backing helped increase the legitimacy of the evaluation process and build trust. By including ‘policy experts’ in the evaluation process, the central health and family planning authority created a trusted probe to help channel information they needed, creating feedback loops and new flows of information, and helping build systemic reflexivity. A willingness on the part of government to accept and use evaluation findings reflects a culture of decision making that, at least in part, accepts imperfect outcomes and attempts to learn from limited inferences to make rapid decisions.

As China’s reforms continue, and as the demands of government change, we can expect to see increasing investment in the evaluation of complex reforms. This will require increases in the capacity of a broad range of institutions to provide support to government, in health and in other domains of social (and other) policy. This is creating new institutional linkages and networks of research institutes, and is likely to create space for methodological innovation. Globally, the importance of managing reforms under conditions of complexity is becoming better understood, including in global health. Practices such as those described in this paper are attempts to deal with this. New approaches with a strong utilization focus and realist perspective may have potential for bridging knowledge gaps in managing rapid changes in complexity.

## Abbreviations

CMO: Context-mechanism-outcome
CNHDRC: China National Health Development Research Center
MCH: Maternal and child health
MoH: Ministry of Health
NHFPC: National Health and Family Planning Commission
TCM: Traditional Chinese medicine
UNICEF: United Nations Children’s Fund

## Footnotes

1 Examples include use of meetings, media, and policy briefings to disseminate practices judged to be good and encourage mutual learning [19], rotation/secondments of government officials [20], and field visits by central officials to inspect policy implementation on the ground.
